# The INFORM (International Framework for Rehabilitation Medics) Project to Strengthen the Medical Specialty

**DOI:** 10.1177/27536351231167482

**Published:** 2023-05-03

**Authors:** Manoj Sivan, Stefano Negrini, Carlotte Kiekens, Fary Khan, Gerard E. Francisco, Francesca Gimigliano

**Affiliations:** 1 British Society of Physical and Rehabilitation Medicine, London, UK; 2 Academic Department of Rehabilitation Medicine, University of Leeds, Leeds, UK; 3 Department of Biomedical, Surgical and Dental Sciences, University ‘La Statale’, Milan, Italy; 4 IRCCS Istituto Ortopedico Galeazzi, Milan, Italy; 5 Physical and Rehabilitation Medicine, IRCCS MultiMedica, Milan, Italy; 6 Director Rehabilitation Royal Melbourne Hospital, Melbourne, Australia; 7 Department of Physical Medicine and Rehabilitation, UTHealth McGovern Medical School, and TIRR Memorial Hermann Hospital, Houston, TX, USA; 8 Department of Mental and Physical Health and Preventive Medicine, University of Campania ‘Luigi Vanvitelli’, Naples, Italy

INFORM is a Task Force of the International Society of Physical and Rehabilitation Medicine (ISPRM) to undertake a collaborative project that aims to unify and strengthen the medical specialty of physicians working in the field of rehabilitation. This effort is run in coordination with the European Society of Physical and Rehabilitation Medicine (ESPRM) and the Physical and Rehabilitation Medicine Section and the Board of the European Union of Medical Specialists (UEMS-PRM).

The medical specialty is named Physical and Rehabilitation Medicine (PRM), or Physical Medicine and Rehabilitation (PM&R), Physiatry, or Rehabilitation Medicine (RM). It relates to applying medical skills to improve functioning and quality of life in individuals with musculoskeletal problems, sports injuries, neurological conditions, cardiorespiratory problems, trauma, chronic pain and other disabling health conditions. The specialty is unique in placing less emphasis on the organ system affected and greater emphasis on whole-person functioning considering impairments in body functions and structures, activity limitations and participation restrictions, personal factors (such as age, culture and beliefs) and environmental factors (such as medical treatments and healthcare support available).^1^

The specialty is officially recognised in the USA, Canada, some South American countries, some Asian countries, Australia, New Zealand and most European countries (all but one in the EU) but is not (yet) present in all countries across the globe. Historically, the specialty originated from different streams that constitute part of it, like manual medicine, exercise therapy, physical agents, or parent medical specialties like rheumatology, neurology or orthopaedics.^2^

The specialty as a whole focuses neither on a specific organ system (eg, neurology) nor a specific age (eg, paediatrics) nor area of care (eg, emergency medicine), but on the optimisation of functioning throughout the continuum of care (from acute to chronic health conditions). This poses some challenges in this modern era of super specialisation. The speciality is not as easily recognised and understood as other medical specialities such as neurosurgery, gastroenterology or orthopaedics. While the European Bodies and other International groups like ISPRM worked in the last 50 years to unify the scope of practice and training requirements for the workforce,^3,4^ there is still a lack of an international uniform framework for the name of specialty.^5,6^ This also leads to confusion regarding influence with regulatory and health policy bodies. Allied health professionals are an integral part of the work environment of physicians in the specialty, even more than in other specialties. The rehabilitation multidisciplinary team includes physiotherapists, occupational therapists, speech and language therapists, orthotists and prosthetists, specialised nurses, psychologists and others. These professionals play a greater role in rehabilitation than other specialties and hence the role of physicians can be relatively diminished with also a lack of clear identity in some countries.

The medical specialty however has several unique attractions. It is based on an excellent conceptual model of health cutting across the boundaries of medicine and therapy and spanning the remits of biology and behavioural sciences. With greater emphasis on daily functioning and well-being, the specialty will undoubtedly expand, gain more recognition and have a bigger impact on patients’ lives. The specialty needs to make its position and outlook clear to colleagues in other specialties, allied health professionals, commissioners and most importantly, patients and carers.

The INFORM project will comprise international surveys, consensus workshops and common frameworks development involving all aspects of the specialty (Figure 1). The project primarily aims to focus on: (1) investigating the feasibility of a universal name of the specialty and medical specialist; (2) standardising the scope of practice as per available international standards and (3) implementing the common minimum ISPRM training curriculum for physicians in training worldwide.

**Figure 1.**
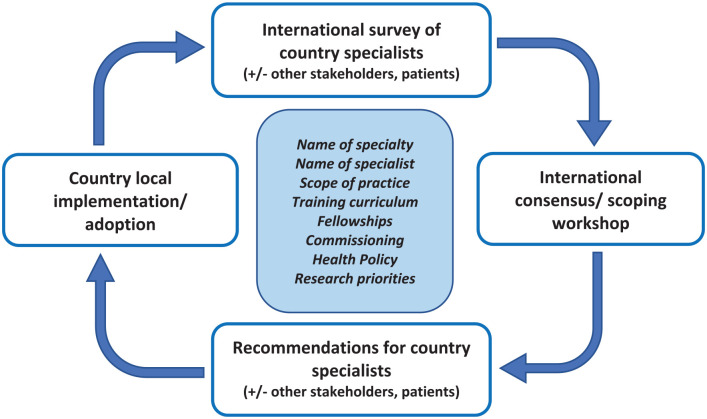
INFORM project conceptual plan.

The project will facilitate an active participatory discussion involving country representatives (members of the task force) to share their views and contribute to consensus statements. The recommendations will then be discussed locally in each country with an aim to adopt as much as possible in their setting. The project task force will help countries in the local implementation phase of the project by helping them address barriers and challenges. There will be active involvement of patients, training regulatory bodies, healthcare policy makers and other organisations which will facilitate the take up of the consensus recommendations locally in each country.

Further details about the project and live results can be found at www.informspecialty.com

## References

[1] Stucki G Cieza A Ewert T , et al. Application of the international classification of functioning, disability and health (ICF) in clinical practice. Disabil Rehabil. 2002;24:281-282. 12004974 10.1080/09638280110105222

[2] European P Rehabilitation Medicine Bodies A. White book on physical and rehabilitation medicine (PRM) in Europe. Chapter 4. History of the specialty: where PRM comes from. Eur J Phys Rehabil Med. 2018;54:186-197. 29565105 10.23736/S1973-9087.18.05147-X

[3] ISPRM. ISPRM Core curriculum and Competencies. 2019. Accessed April 10, 2023. https://isprm.org/isprm-core-curriculum-and-competencies/

[4] EUMS. Training Requirements for the Specialty of Physical and Rehabilitation Medicine. European Standards of Postgraduate Medical Specialist Training. 2018. Accessed April 10, 2023. https://www.uems.eu/__data/assets/pdf_file/0010/64396/UEMS-2018.15-Council-Marrakesh-European-Training-Requirement-PRM-specialty.pdf

[5] Sivan M Haider J Harriss J. Fostering a uniform global name for the specialty of physicians working in rehabilitation. Eur J Phys Rehabil Med. 2022;58:790-792. 36169934 10.23736/S1973-9087.22.07681-X PMC10081480

[6] Negrini S Ferriero G. Neither “What’s in a name?” nor “Nomen omen”, but a unique identifier. A call for a single international name for the speciality and the specialist in PRM, PM&R, RM, Physiatry or other. Eur J Phys Rehabil Med. 2022;58:787-789. 36519584 10.23736/S1973-9087.22.07820-0 PMC10081482

